# Emergence of new lineage of Dengue virus 3 (genotype III) in Lucknow, India

**Published:** 2013-03

**Authors:** Sanjeev Kumar Tripathi, Prashant Gupta, Vineeta Khare, Animesh Chatterjee, Rashmi Kumar, Mohammed Yahiya Khan, Tapan N Dhole

**Affiliations:** 1 Department of Microbiology, Sanjay Gandhi Post Graduate Institute of Medical Sciences, Lucknow, India; 2 Department of Microbiology, King George Medical University, Lucknow, India; 3 Department of Microbiology, Hind Institute of Medical Sciences, Barabanki, India; 4 Department of Pediatrics, King George Medical University, Lucknow, India; 5 Department of Biotechnology, Babasahib Bhimrao Ambedkar University, Lucknow, India

**Keywords:** Dengue virus, phylogenetic analysis, genotype III

## Abstract

**Background and Objectives:**

Dengue has re-emerged as an important arboviral disease causing significant morbidity. It has become hyperendemic in the Indian subcontinent with all four known dengue serotypes circulating.

**Materials and Methods:**

Multiple sequence alignments and phylogenetic trees of DENV-3 were constructed to determine the extent of the isolated dengue virus genetic heterogeneity and phylogeny.

**Results:**

Sequencing and phylogenetic analysis of the C-prM gene junction revealed an active circulation of a new lineage of DENV-3 (genotype III) in this region of India.

**Conclusion:**

Continuous epidemiological surveillance to monitor the incursion and spread of dengue virus genotypes in this region of India is needed.

## INTRODUCTION

Dengue has become a world-wide public health concern and is by far the most important mosquito-borne viral hemorrhagic fever in terms of morbidity, mortality and economic loss (1, 2). It is the most rapidly spreading vector borne disease with around 40% of the global population now living in high risk areas. It is estimated that around 100 million cases of dengue fever (DF) and 500,000 cases of dengue hemorrhagic fever (DHF) occur every year in tropical and subtropical countries, mainly southeast and south Asia, Central and South America, and the Caribbean (3). Rapid urbanization, water storage practices, increase in human and vector populations have offered increased opportunities for the spread of the virus across countries and continents (4–6).

Dengue is caused by four antigenically related, but genetically distinct serotypes designated as dengue virus type 1-4 (DENV 1-4), each of which contains a phylogenetically distinct cluster of viruses referred to as “genotypes” (7–10). Infection with any one of these serotypes generally causes a mild febrile illness, referred to as dengue fever (DF), but some may lead to a severe life threatening disease, called dengue hemorrhagic fever (DHF) or dengue shock syndrome (DSS). Secondary infection is a major risk factor for DHF and DSS through Antibody Dependent Enhancement. Although genotyping is done on the basis of different gene regions, C-prM gene-based genotyping which is capable of amplifying a 511bp region of the C-prM gene of all the four types of dengue virus has been most widely used on Indian isolates and has been established in this study and those carried out by other researchers (11–13).

During the last decade, more frequent and severe epidemics of dengue have hit several Indian cities. The first recorded epidemic of dengue fever in the Asian subcontinent occurred during the 1950s. Until the year 1996, DENV-2 was the predominant serotype circulating in most parts of India. However, three major epidemics struck many parts of the northern India including Delhi, Lucknow and Gwalior in 2003, 2004 and 2006 in which DENV-3 virus was implicated as the major serotype (14–17).

The present study was carried out to investigate the nucleotide sequence diversity in the C-prM gene junction and to determine the phylogeny of DENV-3 that has persistently circulated in Lucknow.

## MATERIALS AND METHODS

### Clinical samples

A total of 298 blood samples from pediatric patients with suspected dengue fever (DF), visiting and admitted in the Institute hospital, were collected during 2006 and 2008. The samples were obtained from different geographical locations within and around the Lucknow city. Informed consent from all the patients was obtained before the collection of clinical samples.

### Serological surveillance

All the serum samples were tested for the presence of dengue specific IgM antibodies by IgM capture ELISA (MAC-ELISA) using a commercial kit (PanBio diagnostic, Brisbane Australia), according to the manufacturer's instructions.

### TaqMan real-time PCR assay

TaqMan real-time one-step RT-PCR amplification was performed with the commercially available Geno-Sen's Dengue 1-4 kit in Rotor gene 6000 (Corbett Research) instrument. The reaction condition were as follows: C-DNA synthesis at 50°C for 15 minutes, an initial denaturation step at 95°C for 10 minutes, 45 cycles of denaturation at 95°C for 15 seconds, annealing at 55°C for 20 seconds, and a final extension step at 72°C for 15 seconds.

### Virus isolation

Isolation of viruses from the acute phase viraemic samples were attempted in C6/36 cells, following the standard virus adsorption protocol as described previously (17). A total of 500 µl of serum samples (diluted 1:10 in PBS) was inoculated onto confluent monolayers of C6/36 cells in 25 cm^2^ tissue culture flask. The inoculum was incubated for 2 hours before being replenished by 10 ml of fresh maintenance media (Eagles minimum essential medium, Sigma) with 2% fetal bovine serum (FBS, Sigma). Uninoculated cell lines were kept for negative control. The cells were then incubated at 32°C and were daily observed microscopically for the appearance of any possible cytopathic effect (CPE). The supernatants of the infected cell culture were collected on the 6^th^-7^th^ day post infection and analyzed for the presence of virus by single step dengue multiplex PCR.

### RNA extraction

Viral RNA was isolated from 140µl of culture supernatant using a QIAmp Viral RNA mini kit (Qiagen, Hidden, Germany) according to the manufacturer's protocol. Final elution was done in 50µl of diethyl pyrocarbonate treated water before storing at -80°C until use.

### Single-step Dengue multiplex RT-PCR (M-RT-PCR)

A one-step single tube serotype-specific multiplex PCR was performed with RNA from viral isolates using a multiplex RT-PCR protocol as described previously (18). The amplification was carried out in a 50 µl total reaction volume with Access quick RT-PCR kit according to the manufacturer's protocol, along with the five primers viz., forward D1 and four serotype specific reverse primers (Ts1, Ts2, Ts3 and Ts4) (Table 1).

**Table 1:** Nucleotide sequences of Dengue virus universal primers (D1, D2), dengue virus serotype specific primers (Ts1, Ts2, Ts3, Ts4).

Primers	Sequence	Base pair	Reference
D1	5’-TCA ATA TGC TGA AAC GCG CGA AAC CG-3’	511 bp	Saxena *et al*, 2008
D2	5’-TTG CAC CAA CAG TCA ATG TCT TCA GGT TC-3’	511 bp	Saxena *et al*, 2008
Ts1	5’-CGT CTC AGT GAT CCG GGG G-3’	482 bp	Saxena *et al*, 2008
Ts2	5’-CGC CAC AAG GGC CAT GAA CAG-3’	119 bp	Saxena *et al*, 2008
Ts3	5’-TAA CAT CAT CAT GAG ACA GAG C-3’	290 bp	Saxena *et al*, 2008
Ts4	5’-CTC TGT TGT CTT AAA CAA GAG A-3’	389 bp	Saxena *et al*, 2008

Briefly, five primers targeting the capsid gene (C) were included in the assay, resulting in different size RT-PCR products of the dengue serotypes (DENV-1, 482 bp, DENV-2, 119 bp, DENV-3, 290 bp, DENV-4, 389 bp).

The thermal profile included a reverse transcription step at 48^°^C for 45 seconds, an initial denaturation at 95^°^C for 2 minutes followed by 35 cycles of denaturation at 94^°^C for 1 minute, annealing at 72^°^C for 1 minute, extension at 72^°^C for 1 minute and a final extension step at 72^°^C for 10 minutes. The PCR amplified products were analyzed on 2% agarose gel using Accu-Prep gel extraction kit (BIO RAD, UK).

### Automated nucleotide sequencing

RT-PCR positive samples were subjected to automated nucleotide sequencing using the BigDye-Terminator-Cycle-Sequencing Ready Reaction kit (Applied Biosystems) as per the manufacturer's protocol.

### Sequence analysis

A BLAST search was carried out to confirm the identity of the strains. Multiple sequence alignment was performed using BioEdit software version 7 employing the Clustal W multiple alignment option (19, 20). The nucleotide sequences identified were translated into amino acid sequences for comparison with other Indian and globally submitted sequences. The DNASTAR software package (Madison, USA) was used to examine the percent identity and diversity among the sequences.

### Nucleotide submission

The five DENV-3 C-prM sequences determined in the present study were submitted to Gen Bank and accession numbers obtained (Table 2).

**Table 2:** Details of Indian DEN-3 isolates sequenced in this study.

S.No	Virus isolate name	Year of collection	Age	Sex	GenBank Accession no.	Pathology
1	DEN/02/UP/INDIA_2006	2006	months 4	M	JF501213	DF
2	DEN/08/UP/INDIA_2006	2006	days 24	M	JF923559	DF
3	DEN/09/UP/INDIA_2006	2006	days 12	M	JF923560	DF
4	DEN/10/UP/INDIA_2006	2006	months 3	F	JF923561	DF
5	DEN/11/UP/INDIA_2006	2006	months 2	F	JF923562	DF

### Phylogenetic tree

Phylogenetic analysis was carried out using MEGA version 3.1 (21). A phylogenetic tree was constructed employing the neighbor-joining method (22) with bootstrap analysis of 1000 replicates (Fig. 2).

**Fig. 1:**
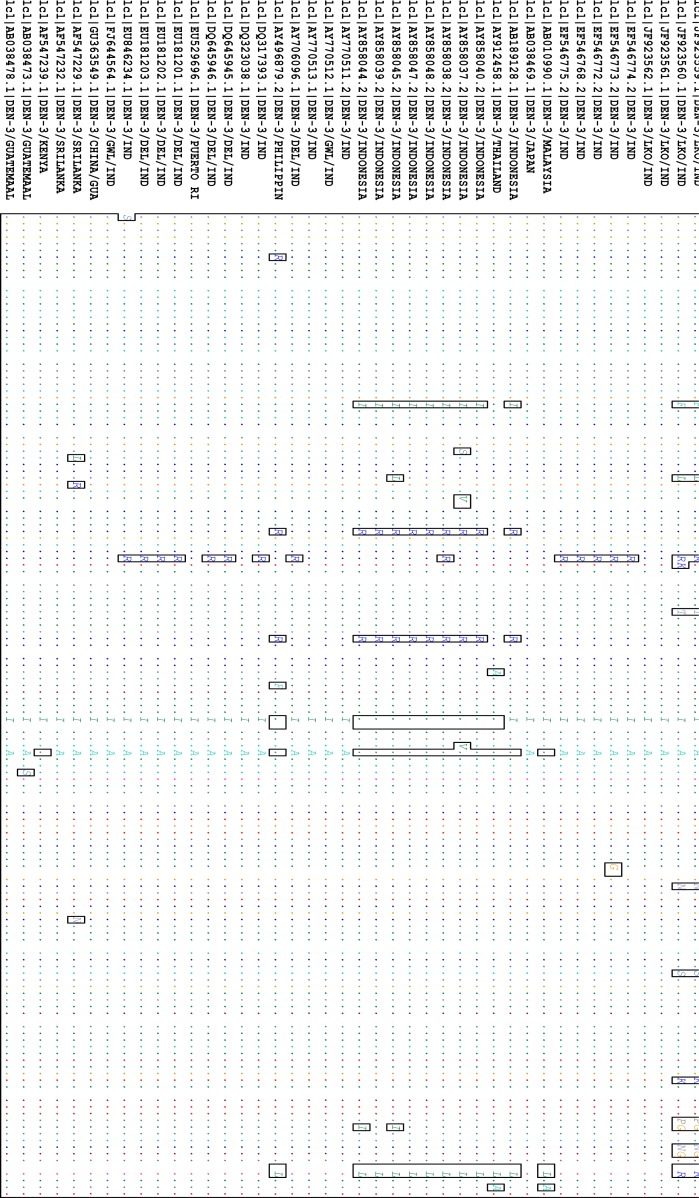
Nucleotide (nt) alignment of CprM gene junction sequences of all Indian and global DEN-3 showing changes in comparison to the consensus sequence. The Indian sequences that are sequenced in this study are the first five strains in the figure. Dot (.) indicates nt homology with the consensus.

## RESULTS

Out of the 298 clinically suspected cases of dengue fever, 66 cases came out to be positive by various methods (IgM MAC ELISA, Viral culture and RT PCR, Real time PCR). Virus culture showed positive CPE in 53 samples which was further confirmed by RT PCR. Multiplex PCR from viral cultures with CPE revealed two serotypes DENV-1 and DENV-3. Of all 53 CPE positive samples 29 were of DENV-1 serotype and 24 were of DENV-3 serotype. Sequencing and phylogeny of 5 DENV-3 viruses was performed. Sequencing and phylogeny of DEN-1 serotypes had been done and published earlier (17).

### Nucleotide sequence and amino acid alignment analysis

The nucleotide sequence of the C-prM gene junction of the five representative dengue viruses determined in the present study were submitted to GenBank and accession numbers obtained (Table 2). An intermediate region of 404 bp (nt 115-518) of the DENV-3 C-prM gene junction was selected for analysis. These sequences were compared and aligned with 18 previously reported Indian sequences and 21 sequences reported from around the world. The alignment did not reveal any base insertion or deletion in this region. Most of the mutations observed in DENV-3 C-prM sequences were transitions which are mostly synonymous in nature. However, some nucleotide changes observed in three Lucknow DENV-3 sequences (JF501213, JF923559, JF923560) were three G > T transversion (nucleotide positions 33, 79 and 310), one G > C transversion (nucleotide position 164), one T > G transversion (nucleotide position 374) and five A>C transversion (nucleotide positions 189, 211, 222, 309 and 370) (Fig. 1). Analysis of the DENV-3 C-prM sequences showed that the Lucknow sequences were closely related to the sequences reported from Guatemala (1998) and presented a nucleotide identity of 92.8%-97.5% (mean 95.15%). Comparing the sequences in this study with other Indian sequences from the years 2003, 2004, 2005, 2006 and 2007, the mean sequence divergence values of 3.75%, 3.75%, 3.70%, 3.50% and 3.70% were observed. Common amino acid mutations observed in the three Lucknow DENV-3 sequences (JF501213, JF923559, JF923560) were valine-phenylalanine (amino acid position 65), serine-leucine (amino acid position 75), lysine-arginine (amino acid position 86), serine-threonine (amino acid position 93), lysine-asparagine (amino acid position 130), alanine-serine (amino acid position 142), glutamic acid-lysine (amino acid position 157), threonine-proline (amino acid position 166), valine-glycine (amino acid position 168) and aspartic acid-asparagine (amino acid position 176).

**Fig. 2:**
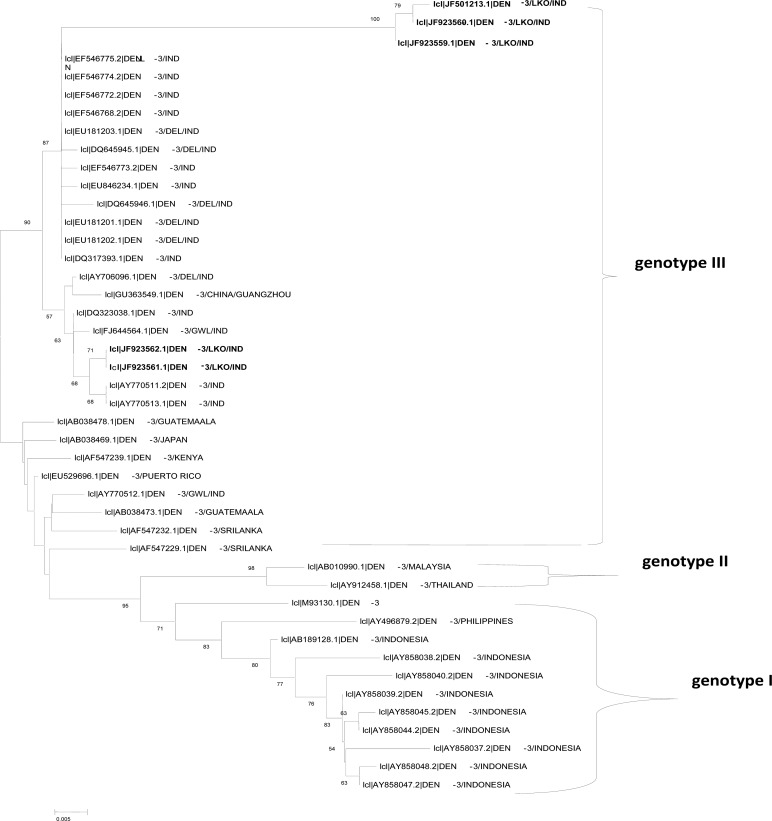
Phylogenetic tree of DENV-3. The tree was generated based on 404 bp region of the CprM gene junction. Strains in the trees are shown by their GeneBank accession number, serotype, place and/ or country of origin. Lucknow strains are shown highlighted in bold.

A phylogenetic tree was constructed using pairwise comparison from the DENV-3 C-prM gene junction of the virus isolates sequenced in this study with the sequences from different parts of the world, which segregated into three genotypes (Fig. 2). All the five DENV-3 strains of the present study were clustered within the genotype III of DENV-3 along with the other previously reported Indian and global sequences from the United States, Caribbean, Africa, Japan and Sri Lanka. All our DENV-3 strains were of genotype 3 yet were distinct from others in this genotype and formed distinct lineage. Genotype II comprised viruses from Malaysia and Thailand isolated in 1994 and 1998, whereas viruses from Philippines and Indonesia were clustered into genotype I. Because of the non-availability of the genotype IV of DENV-3 C-prM sequences, these sequences could not be included while constructing the phylogenetic tree.

## DISCUSSION

Dengue has now re-emerged as the most important arboviral infection in many parts of the south-east Asia including India. This is due to the large scale climatic changes, rapid urbanization and increased frequency of long distance travel by humans that have resulted in the distribution of *Aedes* species in hitherto unknown areas (23). Although, dengue has been endemic in India, all the four known serotypes have been implicated in various outbreaks in the past (11, 24–27), with the major outbreaks being caused by DENV-2. From 2003 onwards, there has been a shift in the cause of these outbreaks from DENV-2 to DENV-3, which has been found to be the predominant dengue virus circulating in the Northern India (11, 25, 26).

In the present study, a molecular epidemiological study of the dengue virus, circulating in the Lucknow city, was carried out using sequence comparison and phylogenetic analysis. Molecular characterization of these viruses is necessary to identify the molecular subtype/genotype and to determine the introduction of any new lineages. Dengue virus genome has three major structural genes; the capsid, pre-membrane and envelope genes along with seven non-structural genes. Different regions of dengue genome have been selected for molecular phylogenetic analysis in the past, but many previous studies have reported the C-prM gene junction as a tool in genotyping the dengue viruses (11, 26, 28). The C-prM gene junction employs a single pair of primers for amplification and sequencing of any of the four serotypes of dengue virus. For phylogenetic analysis, we retrieved the previously reported sequences from the National Centre for Biotechnology Information GenBank database.

On comparison of the sequences, it was found that all the 44 DENV-3 sequences were classified into their respective subtypes/ genotypes. The nucleotide sequencing of all the five isolates of 511 bp amplicon confirmed that the virus sequence was homologous with DENV-3 (genotype III) along with other geographically diverse strains. It was also found that most Indian sequences were very closely related. However, 3 out of the 5 Lucknow strains (JF501213, JF923559, JF923560) formed a different cluster as distinct lineage in phylogenetic tree while two strains were clustered along with the Delhi strains. Although the numbers of isolates selected for sequencing in this study were less yet it is noteworthy that our genotype 3 strains formed a distinct cluster. The possibility of the prevalence of these strains in this region, therefore, cannot be ruled out and thus it could be assumed that the outbreaks that struck fairly large areas of the provinces Lucknow and Delhi, being more than 500 km apart, were actually not caused by the similar type of DENV-3 virus. A single Indian isolate AY770512 from Gwalior was classified into another group, which suggests its different origin from other Indian strains. This study confirms that the circulating Indian DENV-3 strains are similar to the previously reported strains that caused dengue outbreaks in Guatemala and to the other South American genotype III sequences.

It is evident that genotype III of DENV-3 viruses are circulating throughout the world, whereas other genotypes are localized to a particular geographical area. This indicates that the genotype III has a higher potential to disseminate, adapt to and dominate in diverse geographical regions of the world. This genotype has also been implicated in major dengue epidemics from several parts of Asia, Africa and the United States and has the potential to cause an international dengue pandemic (29, 30).

The present study is the first from the region of Lucknow, Uttar Pradesh, which shows that DENV-3 genotype III is circulating in Lucknow region. Similar strains of DENV-3 genotype III have been reported to cause major outbreaks in India (Delhi and Gwalior) and other parts of the world. The re-emergence of the genotype III of DENV-3, replacing the earlier circulating genotype IV of DENV-2 in India, warrants continuous epidemiological surveillance to monitor the incursion and spread of this virus and thus help instructing effective control strategies.
